# The impact of the COVID-19 pandemic on the epidemiology, clinical manifestations and molecular characteristics of *Mycoplasma pneumoniae*

**DOI:** 10.3389/fimmu.2026.1741698

**Published:** 2026-03-16

**Authors:** Luoman Yan, Hengheng Fu, Haiyan Zhang, Hao Dong, Junjie Chen, Lin Yu, Lei Zhang

**Affiliations:** 1 Chengdu Women’s and Children’s Central Hospital, School of Medicine, University of Electronic Science and Technology of China, Chengdu, China; 2 Department of Pediatrics, The Affiliated Hospital of Southwest Medical University, Sichuan Clinical Research Center for Birth Defects, Luzhou, Sichuan, China

**Keywords:** clinical manifestations, epidemiology, macrolide-resistance, molecular characteristics, *Mycoplasma pneumoniae*

## Abstract

*Mycoplasma pneumoniae* (MP) is a major causative agent of acute respiratory tract infections in children. Since 2023, its high prevalence coupled with rising rates of macrolide resistance has presented substantial challenges in the clinical management of pediatric MP infections. In light of the impact of the COVID-19 pandemic on the epidemiology of respiratory pathogens, this article reviews relevant global studies conducted before, during, and after the pandemic. A comprehensive narrative review approach was adopted, with literature searches conducted in databases including PubMed and Web of Science up to December 2025.The findings reveal notable shifts in the epidemiology of MP in the post-pandemic period: the epidemic season has lengthened with a displaced peak, the proportion of cases among school-aged children has risen, and the incidence of severe *Mycoplasma pneumoniae* pneumonia (SMPP) has increased. Globally, macrolide-resistant *Mycoplasma pneumoniae* (MRMP) rates continue to climb, remaining especially high in East Asia (>80%), and are closely linked to specific genotypes such as P1–1 and M4572. Disease severity is associated with both host-derived exaggerated inflammatory responses (e.g., elevated IL-6 and LDH) and the virulence activity of the CARDS toxin. The profile of co-infections has also undergone change. In summary, against a background of reduced pathogen exposure and the formation of immune intervals and high antimicrobial resistance, the COVID-19 pandemic has compounded the clinical complexity of managing MP infections. Future efforts should prioritize enhanced global surveillance, the development of rapid diagnostics and novel therapeutics, and the optimization of antibiotic stewardship strategies.

## 1 Introduction

*Mycoplasma pneumoniae* (MP) is a unique bacterial pathogen lacking a cell wall and represents the most frequent cause of community-acquired pneumonia in children, accounting for approximately 10% to 40% of cases (1). In 2023, an outbreak of MP infections occurred in China, with a prevalence rate as high as 47.89% (2). MP primarily adheres to respiratory epithelial cells via a specialized polar terminal structure and induces pathogenesis through the release of superoxide free radicals and hydrogen peroxide (3). Although MP infection is typically a self-limiting illness with mild clinical manifestations, there has been a growing number of severe or refractory cases in recent years alongside its increased prevalence. Macrolides have been widely used as first-line agents for MP infection and have played a critical role in its treatment. However, extensive macrolide use has led to a rapid increase in macrolide-resistant MP strains, particularly in East Asian countries such as China, Japan, and South Korea, where resistance rates can exceed 90% (4–6). Following the COVID-19 pandemic, shifts in epidemiological patterns have been observed for many respiratory pathogens, including MP (7). This article comprehensively reviews the changes in epidemiological characteristics, clinical manifestations, and molecular types of MP across the pre-, during-, and post-pandemic phases of COVID-19.

## 2 Materials and methods

### 2.1 Literature search strategy

A systematic search was conducted across the PubMed, Web of Science, Embase, and China National Knowledge Infrastructure (CNKI) databases for studies published between January 2017 and December 2025. Search terms included “Mycoplasma pneumoniae,” “COVID-19,” “SARS-CoV-2,” “pandemic,” “epidemiology,” “macrolide resistance,” “molecular typing,” “severe pneumonia,” along with their relevant combinations and corresponding Chinese translations.

### 2.2 Inclusion and exclusion criteria

Studies were included if they: (1) reported on or compared MP infections before, during, or after the COVID-19 pandemic; (2) consisted of original research or high-quality reviews covering at least one of the following aspects: epidemiology, clinical features, drug resistance, or molecular typing; and (3) involved human subjects, with a primary focus on pediatric populations.

Studies were excluded if they: (1) were case reports; (2) were published in languages other than Chinese or English; or (3) had incomplete data or were unavailable in full text.

### 2.3 Literature screening and data extraction

Literature screening and data extraction were performed independently by two researchers through title/abstract screening and full-text review. Any discrepancies were resolved via discussion or adjudication by a third researcher. Data extraction was conducted using a standardized form that included study region, time period, sample size, epidemiological characteristics, macrolide resistance rates, molecular typing information, clinical outcome indicators, and other relevant variables.

### 2.4 Literature quality context

To provide readers with context regarding the robustness of the included evidence, we assessed the methodological quality of all included observational studies using the Newcastle-Ottawa Scale (NOS). The NOS scores ranged from 4 to 9, with a median of 7. The purpose of this assessment was not to perform a formal meta-analysis weighting, but to inform the discussion of evidence heterogeneity and to guide the interpretation of findings. Detailed quality assessment results for each included study are provided in **Supplementary Table 1**.

## 3 The impact of the COVID-19 pandemic on the epidemiological characteristics of MP

### 3.1 Seasonality

MP exhibits an epidemic cycle of approximately 3 to 7 years, with each outbreak typically lasting 1 to 2 years (8). Over the past 15 years, major MP epidemics have been recorded in 2012, 2013, 2019, and 2023. Prior to the COVID-19 pandemic, the primary epidemic season for MP was consistently observed in spring and summer. However, during the later stages of the pandemic, a seasonal shift occurred, with outbreaks extending into autumn and winter and often persisting until the following spring, thereby prolonging the overall epidemic duration (9, 10). Notably, in regions such as Inner Mongolia, China, the epidemic pattern shifted from winter–spring to summer–autumn following the onset of the COVID-19 outbreak (11). However, in Europe and North America, seasonal variation is less distinct, with the primary epidemic seasons occurring in autumn and winter (12, 13).

### 3.2 Susceptible age and gender

The COVID-19 pandemic has influenced not only the seasonal pattern of MP but also the age distribution of susceptible individuals. In the early stages of the pandemic, studies indicated that MP infections primarily affected school-aged children throughout various phases of the epidemic., However, research from Danish scholars later revealed a shift toward older children, with the susceptible age range gradually moving from 5–10 years to 8–12 years. Similarly, studies by Chinese researchers observed a corresponding trend among hospitalized children, noting that the most affected group shifted from infants and toddlers to school-aged children. This shift may be attributed to the lifting of non-pharmaceutical intervention strategies (NPIs), which led to increased mobility, broader social contact, and more indoor gatherings among school-aged children (12, 14).

In contrast, other studies reported a decrease in the median age of MP infection during the later stages of the pandemic, suggesting a trend toward younger susceptible ages. Such discrepancies may be explained by the reduced exposure to MP across all age groups during the NPI period, resulting in lower levels of protective antibodies. As restrictions eased, higher infection rates in adults and older children may have facilitated transmission to infants and young children through close contact. Supporting this, some studies identified identical MLVA genotypes in samples from both children and adults within the same household, indicating possible intra-household transmission of MP (15).

Throughout the pre and post-pandemic periods, the prevalence of MP infection remained consistently higher in female children than in males, with no significant change observed over time (16–18).

### 3.3 Disease spectrum

The implementation of NPIs during the COVID-19 pandemic contributed to a reduction in the incidence of acute respiratory diseases. Throughout the pre- and post-pandemic periods, community-acquired pneumonia (CAP) remained the most frequent respiratory manifestation of MP infection. However, during the pandemic itself, the proportion of CAP and severe pneumonia cases decreased, while diagnoses of bronchiolitis and asthma increased. By 2023, a significant resurgence in CAP and severe pneumonia was observed, accompanied by a decline in asthma and bronchiolitis cases. Notably, the proportion of severe pneumonia cases showed a marked increase (11, 19, 20).

### 3.4 The interplay between pathogen biological traits and delayed resurgence

MP possesses unique biological characteristics, particularly an incubation period lasting 1 to 3 weeks—substantially longer than that of typical respiratory viruses (e.g., 1 to 4 days for influenza virus). This prolonged latency helps explain why other respiratory pathogens rebounded rapidly following the relaxation of pandemic control measures at the end of 2022, whereas a significant resurgence of MP did not occur until 2023. Evidence suggests that the long incubation period and slow transmission dynamics of MP introduce a time lag in its response to NPIs. A recent global modeling study demonstrated that incorporating a three-week latency period and NPI-induced reductions in transmission accurately predicted the observed delay in resurgence. This lag reflects the complex interplay between pathogen biology and the waning of intervention measures—and is key to understanding the post-pandemic “delayed rebound” of MP.

In summary, the COVID-19 pandemic has exerted a substantial influence on the epidemiological profile of MP. Prior to the pandemic, MP exhibited a seasonal pattern, predominantly circulating in spring and summer and primarily affecting school-age children. During the pandemic, NPIs suppressed its transmission, resulting in a delayed epidemic peak and a reduced proportion of severe pneumonia cases. Following the relaxation of control measures in the later phases of the pandemic, a strong resurgence of MP was observed. This rebound was characterized by a notably prolonged epidemic season, shifts in the age distribution of susceptible populations—reported variably as a trend toward either younger or older children—and a significant increase in the proportion of severe pneumonia cases. Despite regional variations, these changes in social behavior induced by the pandemic have ultimately reshaped the epidemiological pattern of MP (**Table 1**).

**Table 1:** Key epidemiological shifts in *M. pneumoniae* infections associated with the COVID-19 pandemic.

Epidemiological feature	Pre-pandemic (up to 2019)	During pandemic (2020-2022)	Post-pandemic (2023 onwards)
Seasonality	Regular peaks in spring/summer; 3–7 year cycles.	Suppressed transmission; delayed/atypical peaks.	Prolonged season (into autumn/winter); displaced peak.
Age Distribution	Primarily school-aged children.	Variable reports: some older (8–12 years), some younger.	Shift reported towards school-aged children; median age debates.
Disease Spectrum	CAP most common; stable proportion of SMPP.	Decreased CAP & SMPP; rise in bronchiolitis/asthma Dx.	Significant resurgence of CAP & SMPP; SMPP proportion increased.
Geographic Notes	Well-described regional patterns in Asia, Europe, US.	Global suppression due to NPIs.	Intense resurgence first noted in Asia (2023), later in Europe/US.

CAP, Community-acquired pneumonia; SMPP, Severe *M. pneumoniae* pneumonia; Dx, Diagnoses.

## 4 The impact of the COVID-19 pandemic on the clinical manifestations

### 4.1 Definitions and terminology

(1) Refractory *Mycoplasma pneumoniae* pneumonia (RMPP) is typically defined as pneumonia in which fever persists and clinical or imaging findings worsen despite at least 7 days of appropriate macrolide antibiotic therapy. (2) Severe *Mycoplasma pneumoniae* pneumonia (SMPP) refers to cases characterized by rapid progression, severe intrapulmonary complications (such as massive pleural effusion, pulmonary necrosis, or respiratory failure), or significant extrapulmonary manifestations. (3) Macrolide-resistant *Mycoplasma pneumoniae* (MRMP) primarily denotes strains carrying point mutations (e.g., A2063G) within domain V of the 23S rRNA gene.

It is important to note that while RMPP/SMPP and MRMP are clinically associated, they represent distinct concepts: the latter refers to a pathogen-related resistance trait, whereas the former describes clinical disease outcomes. The two are therefore not equivalent.

### 4.2 The key pathogenic role of CARDS toxins in serious diseases

Community-acquired respiratory distress syndrome (CARDS) toxin is a key virulence factor of MP. Its production and pathogenic activity are spatiotemporally synchronized with bacterial adhesion—a coordination essential to its central role in severe infections (21, 22). The C-terminal β-trilobed domain of CARDS toxin targets host cell surface receptors (e.g., surfactant protein A, annexin A2) and membrane lipid microdomains, efficiently driving endocytosis (23, 24). Once internalized, the N-terminal mono-ADP-ribosyltransferase domain catalyzes ADP-ribosylation, disrupting critical cellular processes. Concurrently, toxin-induced vacuolation amplifies cellular injury, culminating in widespread cell death and histopathological damage (24, 25). This cascade—spanning targeted adhesion to dual cytotoxic effects (enzymatic and vacuolating)—provides a molecular-pathological foundation for the severe clinical manifestations observed in RMPP/SMPP, including intense inflammatory responses, epithelial barrier disruption, and airway obstruction.

### 4.3 Clinical features

The primary clinical manifestations of *Mycoplasma pneumoniae* pneumonia (MPP) in children are cough and fever, often accompanied by wheezing and tachypnea. Common pulmonary signs include moist rales, wheezing, and diminished breath sounds. Imaging findings frequently consist of lung consolidation, pleural effusion, atelectasis, bronchiectasis, and cavitary lesions (26, 27). Clinically, some studies reported a reduction in extrapulmonary manifestations (e.g., cutaneous and neurological symptoms) in children with MPP after NPIs, but a higher incidence of obstructive airway disease, chest pain, and pleural effusion (28). Conversely, another study observed more frequent extrapulmonary manifestations such as erythema multiforme in the 2023 cohort, with heterogeneous results potentially attributable to regional and prevention and control intensity variations (29).

### 4.4 Immune and inflammatory markers

Early research primarily focused on distinguishing refractory or R/SMPP from general MPP using conventional inflammatory, tissue injury, and coagulation markers, with growing attention to the potential association between MRMP and disease severity. During the early COVID-19 pandemic, children with R/SMPP exhibited significantly higher levels of white blood cell count (WBC), neutrophil percentage (N%), C-reactive protein (CRP), erythrocyte sedimentation rate (ESR), procalcitonin (PCT), and tissue damage markers such as lactate dehydrogenase (LDH), alanine aminotransferase (ALT), and aspartate aminotransferase (AST) compared to those with general MPP (30, 31). D-dimer levels were notably elevated in RMPP patients and positively correlated with CRP, LDH, ESR, pneumonia severity, and pulmonary sequelae (30, 32).

Excessive immune responses, including cytokine storms, are central to severe disease pathogenesis. Serum levels of inflammatory cytokines such as IL-6, IL-8, and TNF-α are significantly elevated in children with SMPP. Moreover, children in the high MP-DNA load and SMPP groups exhibited significantly higher concentrations of IL-6, IL-10, and TNF-α in their bronchoalveolar lavage fluid (BALF) (33), suggesting that excessive inflammation is linked to disease severity and highlighting the central role of a “cytokine storm” in severe illness progression. Additionally, MRMP infection, high MP -DNA load, and co-infection with adenovirus (ADV) are recognized as important risk factors for RMPP and are associated with prolonged fever duration, wheezing, and lung consolidation (34).

During the pandemic, research expanded to include novel serum biomarkers and provided deeper insights into airway-local immune responses and co-infection profiles. Beyond traditional markers such as CRP, LDH, and D-dimer, serum ferritin (SF), fibrinogen (FIB), fibrin degradation products (FDP), neutrophil-to-lymphocyte ratio (NLR), and CK-MB have also been implicated in the development of SMPP/RMPP and may help predict complications such as necrotizing pneumonia (35–37). Studies further indicate that MP-DNA load in BALF positively correlates with inflammatory cytokines including IL-6, IL-10, TNF-α, and IFN-γ, but negatively correlates with CD4+ T-cell counts and the CD4+/CD8+ ratio, confirming intense local inflammation and pulmonary immune imbalance. Cases of airway mucus plug formation have also increased among children with RMPP and MRMP (33, 38). Children with SMPP not only showed elevated CRP, PCT, LDH, and D-dimer, but also exhibited alterations in lymphocyte subsets—marked by reduced CD4+ T-cell counts and CD4+/CD8+ ratios, elevated neutrophils and monocytes, and decreased lymphocytes and eosinophils (39, 40). One study noted that NK cell (CD16+CD56+) levels were significantly higher in the pre-COVID-19 group (2019) than in the post-COVID group (2023), suggesting that NPIs may have altered population immune backgrounds (29). CRP, LDH, D-dimer, and ALT/AST remain reliable indicators for assessing disease severity.

### 4.5 Changes in co-infection patterns

The spectrum of MPP co-infections has also shifted, in the early epidemic stage of the COVID-19, co-infections were observed with both viruses and bacteria; the most frequent viral agents were *adenovirus* (ADV) and *parainfluenza virus*, while *Streptococcus pneumoniae* was the predominant bacterial pathogen (31, 34, 41). During the later stages of the COVID-19 pandemic, with *Streptococcus pneumoniae*, *Haemophilus influenzae*, and *human rhinovirus* (RhV) emerging as the most prevalent co-pathogens (42). *Streptococcus pneumoniae* and *Haemophilus influenzae* remain the most common bacterial pathogens, while *respiratory syncytial virus* (RSV) is the predominant viral co-pathogen in SMPP. *Rhinovirus* (RhV), *parainfluenza virus* (PIV), and *influenza virus* are also frequently detected (39, 43, 44).

**Figure 1:**
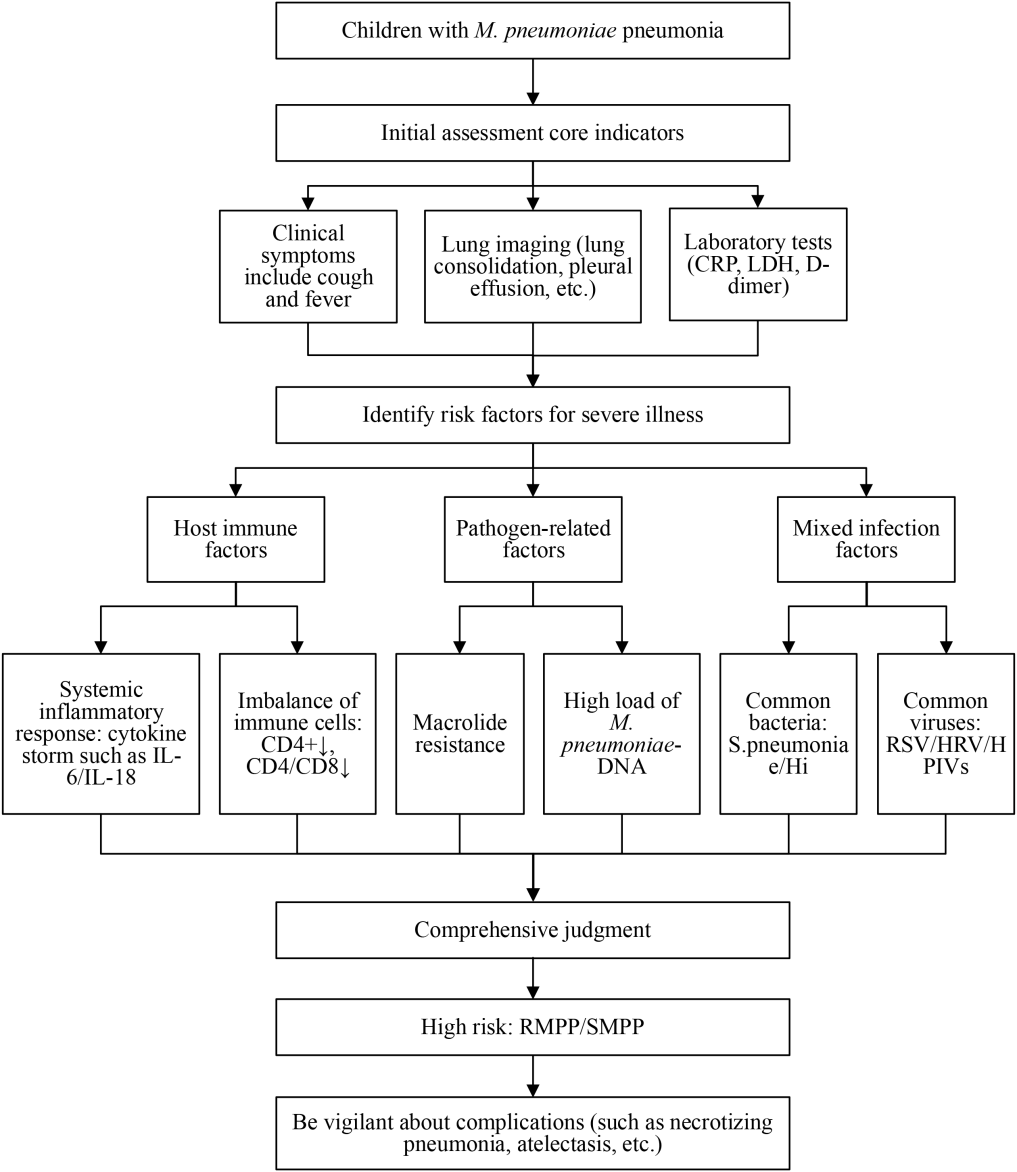
Assessment flowchart for refractory and severe *M. pneumoniae* pneumonia (RMPP/SMPP) and identification of risk factors. This schematic summarizes the initial clinical assessment, key laboratory and imaging indicators, and the multifactorial risk stratification model for pediatric *M. pneumoniae* pneumonia. It integrates host immune factors (e.g., cytokine storm, lymphocyte imbalance), pathogen-related factors (e.g., macrolide resistance, high bacterial DNA load), and co-infection patterns to guide the identification of high-risk patients who may progress to RMPP or SMPP and to alert clinicians to potential complications. Abbreviations: CRP, C-reactive protein; LDH, lactate dehydrogenase; RSV, respiratory syncytial virus; HRV, human rhinovirus; HPIVs, human parainfluenza viruses; Hi, *Haemophilus influenzae*. Conceptual diagram synthesized from the literature reviewed in this article.

The COVID-19 pandemic has significantly changed the clinical presentation of MPP in children. Initially, cases showed typical respiratory symptoms and lung consolidation, with research focusing on markers like CRP and LDH to predict severity. During the pandemic, NPIs altered the disease profile, shifting research toward new biomarkers (e.g., SF, NLR) and mechanisms like local airway immune imbalance. Later, the disease spectrum evolved, with more frequent extrapulmonary manifestations (e.g., erythema multiforme) and increased severity. The pattern of co-infections also changed, shifting from adenovirus and parainfluenza virus to rhinovirus, RSV, *Streptococcus pneumonia* and *Haemophilus influenzae*. NPIs may have also affected population immunity, such as NK cell levels. Consequently, the contemporary understanding of MPP has evolved from a pathogen-centric view to a complex model that integrates host immunity and co-infections, thereby presenting new challenges for clinical management. **Figure 1** delineates the risk assessment process and the multifactorial risk stratification model for RMPP/SMPP (**Table 2**).

**Table 2:** Key biomarkers associated with disease severity in *M. pneumoniae* pneumonia (MPP) and their evolving clinical context.

Biomarker category	Specific biomarkers	Association with RMPP/SMPP	Clinical utility & notes
Conventional Inflammatory & Tissue Injury	CRP, LDH, ESR, PCT, ALT/AST	Significantly elevated.	Core prognostic indicators. LDH and D-dimer strongly correlate with severity and sequelae.
Coagulation Dysfunction	D-dimer, FDP, FIB	Elevated; D-dimer is a independent risk predictor.	Predicts disease severity, complications (atelectasis, necrosis), and pulmonary sequelae.
Novel Serum Biomarkers	Serum Ferritin (SF), NLR, CK-MB	Elevated levels observed.	Emerging role in predicting severe disease and complications like necrotizing pneumonia.
Cytokines (Systemic/Local)	IL-6, IL-8, IL-10, IL-18, TNF-α, IFN-γ	Significantly elevated, especially in BALF.	Indicate “cytokine storm” and exaggerated host immune response. BALF levels correlate with MP-DNA load.
Lymphocyte Subsets	CD4+ T cells ↓, CD4+/CD8+ ratio ↓, NK cells (variable)	Immune cell imbalance is a hallmark.	Reflects systemic immune dysregulation. Changes may be influenced by pandemic NPIs.
Pathogen Load	*M. pneumoniae* DNA load in BALF/serum	High load is a major risk factor.	Positively correlates with inflammation severity and negatively with CD4+ levels. Key for diagnosing RMPP.

RMPP, Refractory MPP; SMPP, Severe MPP; BALF, Bronchoalveolar lavage fluid; NLR, Neutrophil-to-lymphocyte ratio.

### 4.6 Regional differences in clinical heterogeneity: insights from molecular basis and biomarkers

The integration of molecular typing with clinical data reveals that the clinical manifestations of MPP in the later stages of the COVID-19 pandemic exhibited significant regional heterogeneity. This variability can be attributed to differences in circulating genotypes, drug resistance patterns, and host immune backgrounds.

#### 4.6.1 East Asia: a phenotypic profile of high drug resistance and hyperinflammation

The East Asian region is characterized by a persistently high prevalence of MRMP (Chih-Cheng 45). Relevant research indicates that the MP epidemic in China in 2023 was predominantly driven by two drug-resistant clonal strains (T1-2-EC1 and T2-2-EC2). Their evolutionary origins can be traced back to 1997 and 2014, respectively—timelines that closely coincide with the widespread clinical use of azithromycin for pediatric community-acquired pneumonia in China (46). A global meta-analysis further supported these observations, identifying P1-1, 4572, and ST3 as genotypes with the highest resistance rates, all of which are significantly more prevalent in East Asia than in other regions (47).

A study conducted in 2025 first identified a correlation between genotype and clinical severity, showing that children infected with the P1–1 genotype had a higher requirement for respiratory support and mechanical ventilation, suggesting that this genotype may possess enhanced pathogenic potential (48). More recent findings indicate that children over 5 years of age infected with M3562 exhibit significantly elevated levels of CRP and white blood cells (49). In summary, children infected with resistant clones such as ST3, P1-1, and M3562 tend to present with a higher inflammatory burden, reflecting more severe lung parenchymal injury and microcirculatory disturbances. This “high drug resistance–high inflammation” phenotype may be associated with heightened innate immunogenicity of drug-resistant clones.

#### 4.6.2 Europe: a phenotypic profile of low macrolide resistance and pronounced immune response

Contrasting with East Asia, macrolide resistance rates in certain parts of Europe remain relatively low. According to ESGMAC global surveillance, the overall resistance rate in Europe is only 2.02% (50). Nevertheless, a distinctive clinical phenomenon has been observed in this low-resistance setting—namely, an increased incidence of reactive infectious mucocutaneous eruption (RIME). Relevant case studies indicate that RIME may occur in up to 25% of MP infections, and should be considered even in the absence of typical respiratory symptoms (51). Further supporting this observation, a national cohort study from Denmark confirmed that during the MP epidemic in 2023, the proportion of extrapulmonary manifestations—particularly mucocutaneous involvement—was higher among children and adolescents compared to previous outbreaks (52).

Regarding the association between genotype and extrapulmonary manifestations, a study from Slovenia reported that although genotype was not broadly associated with skin involvement, infection with M3662 was significantly associated with severe rashes requiring hospitalization (53). During the 2023 resurgence in Europe, the severity of infections among adults also increased, suggesting that host immune responses may present differently in settings with low antibiotic resistance. This “low resistance–high immune response” phenotype suggests that in the absence of antibiotic selective pressure, the host immune response pattern to susceptible strains may differ fundamentally, manifesting more frequently as immune complex-mediated mucosal injury.

#### 4.6.3 Integrating genotypic and clinical phenotypic data: the biphasic clinical landscape hypothesis

Based on these findings, we propose the “bimodal clinical landscape” hypothesis as a conceptual framework for understanding MP infection patterns in the post-pandemic era. (1) The East Asian phenotype, dominated by the expansion of macrolide-resistant clones (ST3/P1-1/M3562), is distinguished by a hyperinflammatory response, high clinical severity, and a low incidence of classic extrapulmonary manifestations. This phenotype is primarily shaped by intense and prolonged antibiotic selection pressure, exacerbated by the empiric use of azithromycin during the pandemic. (2) The European phenotype, in contrast, is largely attributable to susceptible strains. It is defined by a balanced inflammatory response, a predominance of mild-to-moderate pneumonia, and a higher frequency of immune-complex mediated extrapulmonary manifestations, such as RIME. This profile likely reflects a different host-pathogen interaction dynamic under minimal antibiotic stress.

It should be noted, however, that the direct link between P1 genotype and clinical severity remains inconclusive. Although a 2025 study with a substantial sample size (1,907 cases) failed to demonstrate such an association, global meta-analyses indicate that the statistical power to detect this relationship may have been insufficient across existing studies (47, 54). These discrepancies suggest that the genotype–phenotype correlation is likely influenced by contextual variables such as regional epidemiology, host population, and co-infection status—factors that warrant further investigation through large-scale prospective designs.

This schematic summarizes the initial clinical assessment, key laboratory and imaging indicators, and the multifactorial risk stratification model for pediatric MPP. It integrates host immune factors (e.g., cytokine storm, lymphocyte imbalance), pathogen-related factors (e.g., macrolide resistance, high bacterial DNA load), and co-infection patterns to guide the identification of high-risk patients who may progress to RMPP or SMPP and to alert clinicians to potential complications. Abbreviations: CRP, C-reactive protein; LDH, lactate dehydrogenase; RSV, respiratory syncytial virus; HRV, human rhinovirus; HPIVs, human parainfluenza viruses; Hi, *Haemophilus influenzae*. Conceptual diagram synthesized from the literature reviewed in this article.

## 5 Impact of the COVID-19 pandemic on macrolide resistance trends

MRMP rates have historically remained low to moderate in North America and Europe, though recent surveillance indicates a rising trend. In the United States, a study reported a resistance rate of only 2.8% between 2015 and 2019, suggesting a low risk of encountering MRMP in clinical practice during the early phase of the COVID-19 pandemic (55). Similarly, German data from 2016 to 2018 showed a low resistance rate of 3.0%; however, recent 2023–2024 data reveal an increase to 14%, indicating a potential emergence of macrolide-resistant strains in Europe (56, 57). By contrast, a Russian study reported a macrolide resistance rate of 40% in 2023–2024—significantly higher than that in Western Europe and North America—highlighting Eastern Europe as a region with moderate-to-high resistance (58). Compared with these regions, Asia—particularly East Asia—has experienced the highest and most dynamic shifts in MRMP prevalence. In China, MRMP rates have shown a continuous upward trajectory, rising from 46.74% in the pre-pandemic period, to 72.63% during the pandemic, and further to 83.31% in the post-pandemic phase (59). Japanese data revealed considerable fluctuation, with resistance rates varying from 56.3% to 24.6% between 2015 and 2020, followed by a rebound to approximately 47.9% after 2023. This non-linear trend suggests that resistance may be influenced by multiple factors, including shifts in circulating strains and changes in antibiotic policies (60–62). In South Korea, MRMP has remained persistently high. While the rate decreased slightly from an extreme level of 84.4% during 2014–2016 to 69.67% in 2019–2020—potentially due to non-pharmaceutical interventions—the most recent 2023 data show a resurgence to 87%, underscoring the ongoing challenge of high macrolide resistance in the region. (63–65).

### 5.1 Selective pressure of macrolide use and clonal expansion of drug-resistant strains

It is notable that during the COVID-19 pandemic, azithromycin was widely used as an experimental treatment drug for COVID-19 worldwide, even in the absence of clear evidence of bacterial infection (66). This unconventional use of antibiotics may exert a strong selective pressure on the emergence of macrolide-resistant strains, accelerate the expansion of drug-resistant clone strains (such as ST3 type), and this large-scale use of antibiotics may also reshape the drug-resistant ecosystem of respiratory pathogens. Especially in East Asia, the limited selectivity and usage habits of antibiotics may have magnified this effect, which can also explain why the resistance rate of MP in East Asia has remained consistently above 80%. MRMP epidemiology varies markedly by region, reflecting local antibiotic use and circulating strains. Resistance rates are not static and appear to be influenced by a range of local and temporal factors, leading to divergent trends across different regions (**Table 3**).

**Table 3:** Global trends in macrolide-resistant *M. pneumoniae* (MRMP) rates before, during, and after the COVID-19 pandemic.

Region/country	Pre-pandemic (to 2019)	During pandemic (2020-2022)	Post-pandemic (2023-2024)	Key references & notes
Asia	~			Highest endemic area.
China	~46-84%	~73%	~83% (rising trend)	11, 59
Japan	~56-90%	~25-56% (fluctuating)	~48% (rebound)	60–62
South Korea	~69-84%	~70%	~87% (persistently high)	63–65
Europe				Historically low, now rising.
Germany	~3%	Data limited	~14% (notable increase)	56, 57
Russia	Data limited	Data limited	~40% (moderate-high)	58
North America				Remains relatively low.
United States	~2.8%	Data limited	Recent data scarce; likely still low-moderate	55

## 6 The influence of the COVID-19 pandemic on the molecular types of MP

MP can be classified into different genotypes using methods such as P1 typing, multilocus variable-number tandem repeat analysis (MLVA), and multilocus sequence typing (MLST) (67, 68). The distribution of prevalent genotypes varies across geographic regions and ethnic groups and evolves over time, influenced by factors such as racial background and local environmental conditions. Among P1 genotypes, type I strains demonstrate a higher propensity for point mutations compared to type II and are associated with an increased risk of SMPP and extrapulmonary complications (69).

In the early phase of the COVID-19 pandemic, the P1–1 genotype was dominant in China and other countries (e.g., Sweden and Southeastern Finland) (70, 71), accounting for over 75% of isolates in most studies. However, regional variations were observed, for instance, in Nanjing, the P1–2 type was the predominant strain, highlighting the influence of geographic factors on genotype distribution (61, 67, 72). The M4572 genotype, once the most prevalent MLVA type in China and other countries (e.g., Sweden and Southeastern Finland) (70, 71), previously linked to macrolide resistance, showed a gradual decline in prevalence during this period. Interestingly, its resistance rate did not appear to be strongly influenced by prior macrolide exposure (67, 72–74). Concurrently, the M3562 genotype increased in frequency, rising from 60% in 2016 to 93.48% in 2019—a trend that may be associated with the rising rate of macrolide resistance (73). In MLST-based classifications, ST3 has been frequently identified among MRMP isolates and has progressively become a dominant sequence type (60, 75).

In the later stages of the pandemic, following the relaxation of non-pharmaceutical interventions (NPIs), MP infections resurged markedly, accompanied by a further increase in macrolide resistance. Molecular characterization revealed both continuity and intensification of pre-existing evolutionary trends. Among P1 genotypes, P1–1 remained dominant and even strengthened its prevalence: in Beijing, it accounted for 76.1% of samples in 2023 (39), while in Suzhou, it reached 81.4% (76). Similarly, the M4572 MLVA type rebounded from its earlier decline, appearing in 70%–90% of samples across multiple studies (39, 76, 77). Previous studies in Japan have suggested that the dominant strain replacement cycle for MP is approximately 10 years (78). The re-emergence and consolidation of M4572 in China may reflect disruption of this natural cycle due to widespread NPIs during the pandemic.

**Figure 2:**
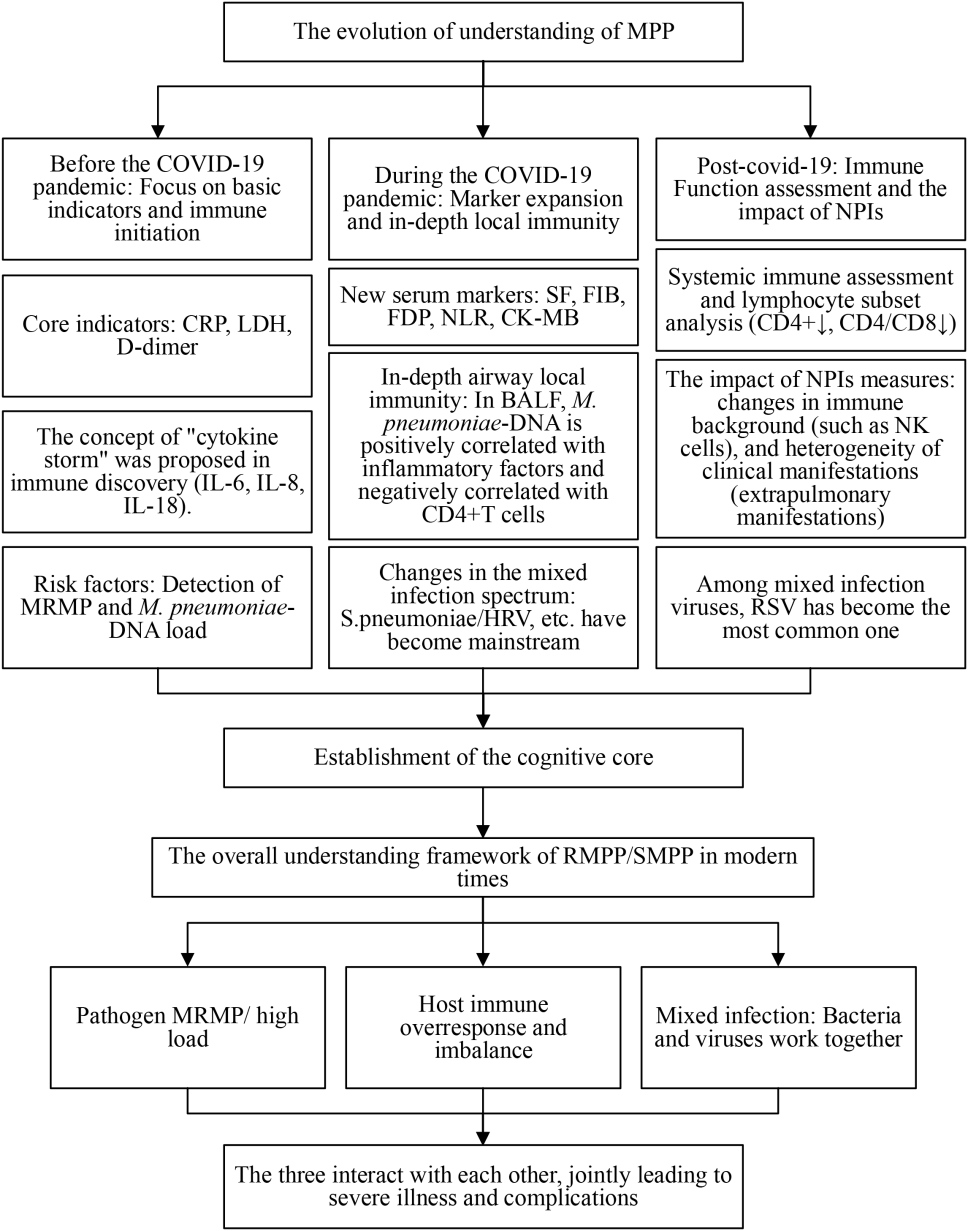
Evolution in the understanding of *M. pneumoniae* pneumonia (MPP) and the immunological mechanisms of severe disease. The timeline illustrates the shifting research focus and expanding knowledge framework of MPP across the pre-, during-, and post-COVID-19 pandemic eras. It highlights the progression from foundational clinical and basic inflammatory markers to the exploration of novel serum biomarkers, localized airway immunity, systemic immune profiling, and the impact of non-pharmaceutical interventions (NPIs). The modern understanding conceptualizes severe disease (RMPP/SMPP) as the result of a complex interplay between host immune overresponse/dysregulation, pathogen-specific factors (MRMP, high load), and synergistic co-infections. Diagram based on the synthesis of findings from studies included in this review.

Pre-pandemic genotypes P1–1 and M4572 were initially dominant. Following the widespread use of NPIs, natural transmission and strain replacement cycles were suppressed. After NPIs were relaxed, infections resurged with a strong rebound and consolidation of the P1–1 and M4572 genotypes, suggesting the pandemic measures disrupted the pathogen’s typical evolutionary dynamics. (**Table 4**) As illustrated in **Figure 2**, our understanding of MPP and its severe disease mechanisms has evolved significantly across the pandemic, culminating in a comprehensive framework that integrates host, pathogen, and co-infection factors.

**Table 4:** Predominant molecular genotypes of *M. pneumoniae* and their temporal shifts in the context of the COVID-19 pandemic.

Genotyping method	Predominant type(s) pre-/early pandemic	Shift & predominant type(s) post-pandemic (2023-)	Association with severity/resistance	Notes
P1 Type	P1–1 dominant in most areas (e.g., China, Japan, Sweden). P1–2 dominant in some regions (e.g., Nanjing).	P1–1 dominance strengthened and consolidated (e.g., >75-81% in China).	Type I (P1-1) associated with higher risk of SMPP and extrapulmonary complications.	Geographic variation persists; pandemic NPIs may have disrupted natural strain replacement cycles.
MLVA Type	M4572 (previously dominant, then declining). M3562 (increasing, linked to rising MRMP).	M4572 rebounded strongly (70-90% in multiple Chinese studies).	M4572 historically linked to macrolide resistance. M3562 increase correlated with MRMP rise.	Re-emergence of M4572 post-NPIs is a notable feature of the post-pandemic resurgence.
MLST	ST3 frequently identified among MRMP isolates.	ST3 remains a dominant sequence type among MRMP.	ST3 strongly associated with macrolide-resistant phenotypes.	Clonal expansion of resistant ST3 strains reported in East Asia.

MLVA, Multilocus variable-number tandem repeat analysis; MLST, Multilocus sequence typing.

The timeline illustrates the shifting research focus and expanding knowledge framework of MPP across the pre-, during-, and post-COVID-19 pandemic eras. It highlights the progression from foundational clinical and basic inflammatory markers to the exploration of novel serum biomarkers, localized airway immunity, systemic immune profiling, and the impact of non-pharmaceutical interventions (NPIs). The modern understanding conceptualizes severe disease (RMPP/SMPP) as the result of a complex interplay between host immune overresponse/dysregulation, pathogen-specific factors (MRMP, high load), and synergistic co-infections. Diagram based on the synthesis of findings from studies included in this review.

## 7 Discussion

This narrative review synthesizes the evolving landscape of MP infections before, during, and after the COVID-19 pandemic. Several key themes emerge from our analysis.

### 7.1 The delayed resurgence: a consequence of pathogen biology

Our finding that MP resurgence exhibited a delayed trajectory compared to other respiratory pathogens (Section 3.4) underscores the critical role of pathogen-specific biological traits in shaping post-pandemic epidemiological patterns. While the “immune debt” hypothesis provides a unifying explanation for increased population susceptibility across pathogens, the uniquely prolonged incubation period of MP (1–3 weeks) introduced a temporal lag in its response to the removal of non-pharmaceutical interventions (NPIs). This observation carries practical implications: surveillance systems must account for such pathogen-level differences when forecasting post-intervention epidemic rebounds.

### 7.2 Regional heterogeneity: beyond the immune debt hypothesis

Perhaps the most striking finding is the marked dichotomy between East Asian and European clinical phenotypes (Section 4.6). The East Asian “high-resistance, high-inflammation” phenotype—predominantly driven by ST3/P1-1/MLVA-3562 clones and characterized by elevated LDH and D-dimer levels—reflects decades of intensive macrolide selection pressure, further amplified by off-label azithromycin use during the COVID-19 pandemic (46, 66). In contrast, the European “low-resistance, high-immune response” phenotype, marked by an increased incidence of RIME (52), suggests that in the absence of sustained antibiotic pressure, host immune responses may target distinct pathogenic pathways. This regional dichotomy cannot be fully explained by the “immune debt” hypothesis alone; rather, it necessitates an integrated framework that accounts for antibiotic selection pressure, clonal expansion dynamics, and host immune background.

### 7.3 Genotype–phenotype correlations: a work in progress

The association between MP genotypes and clinical severity remains controversial. While some studies have reported links between the P1–1 genotype and severe outcomes (48), others have failed to replicate such findings (54). Global meta-analyses suggest that prior studies may have been underpowered to detect significant associations (47). These inconsistencies likely reflect the multifactorial nature of disease severity, which is modulated by host factors, pathogen characteristics, and environmental variables. Future investigations should adopt standardized definitions and integrate multi-omics approaches to disentangle the complex interplay underlying genotype–phenotype correlations.

### 7.4 Limitations and prospects

Studies in this field often face inherent limitations, including single-center designs and relatively small sample sizes. Single-center studies are typically restricted to specific geographic regions and patient populations, which may compromise external validity and necessitate caution when generalizing findings to other settings or broader populations. In addition, limited sample sizes not only reduce statistical power but also diminish the precision of effect estimates. Collectively, these scale-related constraints weaken the overall strength of the evidence and limit its suitability as a foundation for high-level scientific support.

Furthermore, the availability of data from Europe and North America remains limited. The inclusion of studies with such geographic constraints may introduce heterogeneity into the overall findings. As a result, the conclusions drawn from this body of literature should be regarded as exploratory or provisional rather than confirmatory. It is also important to note that this article adopts a narrative review approach rather than a systematic review strictly following PRISMA guidelines. Although a systematic literature search and quality assessment were conducted, we did not perform a quantitative meta-analysis or provide a PRISMA flowchart. Consequently, there may be a degree of selection bias in the literature screening process.

Future research should aim to address these limitations through well-designed multicenter studies, large-sample clinical trials, or prospective cohort studies. Such efforts will help generate more generalizable and definitive evidence to advance understanding in this field.

## 8 Conclusion

In summary, the heightened clinical complexity of post-pandemic MP infection reflects a multifactorial interplay: the global expansion of hyperinflammatory, drug-resistant clones (e.g., ST3/P1-1/M3562); an altered host immune landscape resulting from pandemic-related non-pharmaceutical interventions and the accumulation of “immune debt”; and the sustained selective pressure exerted by widespread macrolide use, including off-label prescribing during the COVID-19 crisis. The convergence of pathogen evolution, host immune modulation, and antibiotic selection pressure has culminated in the current biphasic clinical landscape: a hyperinflammatory, resistant phenotype in East Asia and an immune-driven, susceptible phenotype in Europe. This paradigm necessitates a shift from a pathogen-centered approach to a holistic “pathogen–host–environment” framework. Future efforts must integrate global resistance surveillance with molecular typing (P1/MLVA) and biomarker-guided strategies to enable precision therapeutics.
