# Supplementary material for: The impact of the COVID-19 pandemic on the epidemiology, clinical manifestations and molecular characteristics of Mycoplasma pneumoniae
Source: Front Immunol. 2026 Mar 16;17:1741698. doi: 10.3389/fimmu.2026.1741698 (PMC13033506; doi:10.3389/fimmu.2026.1741698)
Supplement: Supplementary file 1 [file Table1.docx]

**Supplementary Table S1. Literature quality context of included observational studies using the Newcastle-Ottawa Scale (NOS)**

| First author | Year | Study design | Selection (max 4) | Comparability (max 2) | Outcome (max 3) | Total score (max 9) | Quality rating |
| --- | --- | --- | --- | --- | --- | --- | --- |
| Bolluyt DC | 2024 | Cross-sectional | 3 | 1 | 2 | 6 | Moderate |
| Chen B | 2024 | Cross-sectional | 3 | 1 | 2 | 6 | Moderate |
| Chen J | 2025 | Cohort | 4 | 2 | 3 | 9 | High |
| Chen Y | 2024b | Cross-sectional | 4 | 1 | 2 | 7 | High |
| Chen Y | 2018 | Cohort | 3 | 2 | 2 | 7 | High |
| Cheng J | 2025 | Cohort | 4 | 2 | 3 | 9 | High |
| Deng F | 2023 | Cohort | 4 | 2 | 3 | 9 | High |
| Ding W | 2025 | Retrospective cohort | 4 | 2 | 2 | 8 | High |
| Du H | 2024 | Multicenter cohort | 4 | 2 | 3 | 9 | High |
| Dumke R | 2024 | Surveillance | 3 | 1 | 2 | 6 | Moderate |
| Dumke R | 2019 | Surveillance | 3 | 1 | 2 | 6 | Moderate |
| Dungu KHS | 2024 | Nationwide cohort | 4 | 2 | 3 | 9 | High |
| ESGMAC | 2025 | Global surveillance | 4 | 2 | 3 | 9 | High |
| Fan L | 2017 | Cohort | 3 | 2 | 2 | 7 | High |
| Fang KN | 2019 | Cohort | 3 | 2 | 2 | 7 | High |
| Gu W | 2025 | Cohort | 4 | 2 | 3 | 9 | High |
| Gullsby K | 2019 | Surveillance | 3 | 1 | 2 | 6 | Moderate |
| Huang MX | 2022 | Cohort | 4 | 2 | 2 | 8 | High |
| Huang X | 2021 | Cohort | 4 | 2 | 3 | 9 | High |
| Jiang TT | 2023 | Cohort | 4 | 2 | 2 | 8 | High |
| Kenri T | 2020 | Surveillance | 3 | 1 | 2 | 6 | Moderate |
| Kenri T | 2023 | Surveillance | 3 | 1 | 2 | 6 | Moderate |
| Korneenko E | 2025 | Cross-sectional | 3 | 1 | 2 | 6 | Moderate |
| Kurkela S | 2019 | Outbreak investigation | 3 | 1 | 2 | 6 | Moderate |
| Kutty PK | 2019 | Multicenter cohort | 4 | 2 | 3 | 9 | High |
| Lanata MM | 2021 | Cohort | 4 | 2 | 3 | 9 | High |
| Lee E | 2020 | Cohort | 4 | 2 | 2 | 8 | High |
| Lee JK | 2018 | Cohort | 4 | 2 | 3 | 9 | High |
| Lee JK | 2024 | Cohort | 4 | 2 | 3 | 9 | High |
| Li C | 2025a | Cohort | 4 | 2 | 2 | 8 | High |
| Li D | 2023a | Cohort | 4 | 2 | 2 | 8 | High |
| Li S | 2025b | Cross-sectional | 4 | 1 | 2 | 7 | High |
| Li YT | 2023b | Cohort | 3 | 2 | 2 | 7 | High |
| Mao J | 2025 | Cohort | 4 | 2 | 2 | 8 | High |
| Miao Y | 2025 | Large cohort | 4 | 2 | 3 | 9 | High |
| Morozumi M | 2020 | Surveillance | 3 | 1 | 2 | 6 | Moderate |
| Murata M | 2025 | Cohort | 3 | 2 | 2 | 7 | High |
| Nordholm AC | 2024 | Surveillance | 3 | 1 | 2 | 6 | Moderate |
| Qi J | 2025 | Longitudinal | 4 | 2 | 2 | 8 | High |
| Qiu W | 2024 | Cohort | 4 | 2 | 2 | 8 | High |
| Raghuram A | 2025 | Surveillance | 3 | 1 | 2 | 6 | Moderate |
| Rivaya B | 2020 | Cohort | 3 | 2 | 2 | 7 | High |
| Robinson E | 2025 | Retrospective cohort | 4 | 2 | 2 | 8 | High |
| Rodman Berlot J | 2022 | Cohort | 4 | 2 | 3 | 9 | High |
| Rodman Berlot J | 2023 | Cohort | 4 | 2 | 3 | 9 | High |
| Shin S | 2023 | Surveillance | 3 | 1 | 2 | 6 | Moderate |
| Wang F | 2024a | Cohort | 4 | 2 | 2 | 8 | High |
| Wang N | 2022 | Cross-sectional | 3 | 1 | 2 | 6 | Moderate |
| Wang Y | 2021 | Cohort | 4 | 2 | 2 | 8 | High |
| Wen J | 2021 | Case-control | 3 | 2 | 2 | 7 | High |
| Wu TH | 2024 | Cohort | 4 | 2 | 2 | 8 | High |
| Wu TH | 2021 | Cohort | 4 | 2 | 2 | 8 | High |
| Xi Z | 2024 | Cohort | 4 | 2 | 2 | 8 | High |
| Xu L | 2024 | Longitudinal | 4 | 2 | 2 | 8 | High |
| Xue G | 2018 | Cross-sectional | 3 | 1 | 2 | 6 | Moderate |
| Yan C | 2025 | Genomic epidemiology | 4 | 2 | 3 | 9 | High |
| You H | 2025 | Metagenomic study | 4 | 2 | 3 | 9 | High |
| You J | 2024 | Cohort | 4 | 2 | 2 | 8 | High |
| Zhan XW | 2022 | Cohort | 4 | 2 | 2 | 8 | High |
| Zhang J | 2025a | Longitudinal | 4 | 2 | 2 | 8 | High |
| Zhang Y | 2025b | Multicenter cohort | 4 | 2 | 3 | 9 | High |
| Zhao F | 2019 | Cross-sectional | 3 | 1 | 2 | 6 | Moderate |
| Zheng XX | 2020 | Cohort | 3 | 2 | 2 | 7 | High |
| Zhou Y | 2020 | Cohort | 4 | 2 | 2 | 8 | High |
| Zhu X | 2024 | Cohort | 4 | 2 | 2 | 8 | High |

**Notes:**

**Quality rating**: High (≥7), Moderate (5-6), Low (≤4)

**NOS scoring criteria**: Selection: Representativeness of exposed cohort, selection of non-exposed cohort, ascertainment of exposure, demonstration that outcome was not present at start

Comparability: Control for most important factor (1 point) and additional factors (1 point)

Outcome: Assessment of outcome, follow-up length, adequacy of follow-up

Systematic reviews, meta-analyses, case reports, and molecular studies without clinical outcome data were not assessed with NOS.

**Summary of quality distribution**

| Quality rating | Number of studies | Percentage |
| --- | --- | --- |
| High (≥7) | 44 | 71.0% |
| Moderate (5-6) | 18 | 29.0% |
| Low (≤4) | 0 | 0% |

**Total included observational studies assessed: 62**
